# Convergence of Cortical, Thalamocortical, and Callosal Pathways during Human Fetal Development Revealed by Diffusion MRI Tractography

**DOI:** 10.3389/fnins.2017.00576

**Published:** 2017-11-01

**Authors:** Rongpin Wang, Molly Wilkinson, Tara Kane, Emi Takahashi

**Affiliations:** ^1^Division of Newborn Medicine, Department of Medicine, Boston Children's Hospital, Harvard Medical School, Boston, MA, United States; ^2^Department of Radiology, Guizhou Provincial People's Hospital, Guiyang, China; ^3^Department of Behavioral Neuroscience, Northeastern University, Boston, MA, United States

**Keywords:** thalamocortical, corticothalamic, corpus callosal, development, fetus, human, tractography

## Abstract

There has been evidence that during brain development, emerging thalamocortical (TC) and corticothalamic (CT) pathways converge in some brain regions and follow each other's trajectories to their final destinations. Corpus callosal (CC) pathways also emerge at a similar developmental stage, and are known to converge with TC pathways in specific cortical regions in mature brains. Given the functional relationships between TC and CC pathways, anatomical convergence of the two pathways are likely important for their functional integration. However, it is unknown (1) where TC and CT subcortically converge in the human brain, and (2) where TC and CC converge in the cortex of the human brain, due to the limitations of non-invasive methods. The goals of this study were to describe the spatio-temporal relationships in the development of the TC/CT and CC pathways in the human brain, using high-angular resolution diffusion MR imaging (HARDI) tractography. Emerging cortical, TC and CC pathways were identified in *postmortem* fetal brains ranging from 17 gestational weeks (GW) to 30 GW, as well as *in vivo* 34–40 GW newborns. Some pathways from the thalami were found to be converged with pathways from the cerebral cortex as early as 17 GW. Such convergence was observed mainly in anterior and middle regions of the brain until 21 GW. At 22 GW and onwards, posterior pathways from the thalami also converged with cortical pathways. Many CC pathways reached the full length up to the cortical surface as early as 17 GW, while pathways linked to thalami (not only TC axons but also including pathways linked to thalamic neuronal migration) reached the cortical surface at and after 20 GW. These results suggest that CC pathways developed earlier than the TC pathways. The two pathways were widespread at early stages, but by 40 GW they condensed and formed groups of pathways that projected to specific regions of the cortex and overlapped in some brain regions. These results suggest that HARDI tractography has the potential to identify developing TC/CT and CC pathways with the timing and location of their convergence in fetal stages persisting in postnatal development.

## Introduction

Thalamocortical/corticothalamic (TC/CT) and corpus callosal (CC) pathways are major white matter tracts in the brain which are conserved across species throughout evolution, and partake in fundamental brain functions. The TC/CT system is important for consciousness (Bagshaw et al., 2014), working memory (Sarnthein et al., 2005), sensory relay (Jones, 2007), executive control, and salience processing (Seeley et al., 2007). The CC pathway connects the left and right hemispheres and has multiple functional integrational roles in visual processing (Bocci et al., 2014), motor movement (Fabri and Polonara, 2013), and somatosensory processing (Fabri et al., 1999; Marcano-Reik and Blumberg, 2008; Magnuson et al., 2014).

During brain development, the TC axons go through a number of steps in order to reach their final destinations (Rakic, 1975, 1988), following a distinct pathway stemming from the thalamus and reaching into the cerebral cortex (López-Bendito and Molnar, 2003; Garel and Rubenstein, 2004). Around the second and third gestational weeks (GW) in humans, the neocortex and thalamus start to link with each other through specific and reciprocal connections (Leyva-Díaz and López-Bendito, 2013), and continue increasing the number of connections between them. It is still under debate how human TC pathways find a way to go through brain regions to finally project to the cortex.

The “handshake hypothesis” was proposed by Blakemore and Molnár (1990) to explain how TC pathways ascending through the internal capsule project to their cortical targets with assistance from reciprocal descending cortical pathways (Molnár et al., 1998). Métin and Godement (1996) used mice and hamsters and suggested that the ganglionic eminence (GE) may be an intermediate target in the pathfinding of axons between the cortex and the thalamus.

Anatomically, while the TC pathways project to a wide range of areas throughout the brain including visual, auditory, motor, and somatosensory areas, the CC pathways are known to project to only specific regions. For example, in the primary visual cortex, the CC pathways project specifically to the border of V1 and V2 areas, in mice (Wang et al., 2007) and monkeys (Dehay et al., 1986; Kennedy et al., 1986; Dehay and Kennedy, 1988), while the TC pathways project to more broad areas throughout the primary visual cortex, with most connecting to layer IV (Robertson, 1976; Killackey and Belford, 1979; Burton and Kopf, 1984; Afifi and Bergman, 1997; Van Essen, 2005; Viaene et al., 2011). Given the functional relationships between the TC and CC pathways, anatomical convergence of the two pathways at the regions where CC pathways are projecting should be important for their functional integration. However, it is still unknown where these two pathways converge in the human brain due to the limitation of spatial resolution of non-invasive methods. Moreover, their anatomic relationships in more anterior brain regions (other than the visual area) and their spatial relationships during fetal developmental stages are elusive, even in animal brains.

Previous diffusion tensor MR imaging (DTI) tractography studies have been done on the TC (e.g., Johansen-Berg et al., 2005; Yamada et al., 2007; Jaermann et al., 2008; Hong et al., 2010; Klein et al., 2010; Zhang et al., 2010) and CC tracts (e.g., Häberling et al., 2011; Luders et al., 2012; Whitehead et al., 2014), with regionally differential developmental courses (Gilmore et al., 2007). High-angular resolution diffusion MR imaging (HARDI) tractography enables identification of water diffusivity in many different directions in each imaging voxel, which theoretically provides an advantage over DTI (Frank, 2002; Tournier et al., 2004, 2007). HARDI theoretically allows reconstruction of complex crossing tissue coherence in the brain (Tuch et al., 2003) and even in immature fetal brains (e.g., Takahashi et al., 2012), which are typically more challenging to segment due to a surplus of unmyelinated pathways. Although, past diffusion tractography studies were able to detect overall patterns of trajectories of TC and CC pathways in adults and pediatric populations (Jaermann et al., 2008; Lebel et al., 2010; Vasung et al., 2011; Poh et al., 2015), and our past study successfully revealed potential neuronal migration pathways of thalamic neurons and TC development in fetal brains (Wilkinson et al., 2016), there has been no report that aimed to identify convergence of TC/CT pathways and convergence of TC/CC pathways in human fetuses.

The goals of this study were to describe the spatio-temporal relationships of the development of the TC/CT and TC/CC pathways in human fetal brains, using HARDI tractography. One of our hypotheses based on the literature is that TC projections contact with pathways from the cortex. This “handshaking” has been proposed to be seen within the GE, and we specifically analyzed such convergence of TC and cortical pathways within the GE. Another hypothesis in this study is that the convergence of TC and CC pathways in the cortex can be detected with HARDI. We expected that TC and CC pathways converge in specific cortical regions, based on the knowledge that TC pathways project to broad areas in the brain but CC pathways only project to the borders of visual areas.

## Materials and methods

### Postmortem fetal brain specimens and *in vivo* subjects

The data of nine postmortem fetal brains were used in this study. Six postmortem brains (one 17, two 18, and three 20 GW) were obtained from the Department of Pathology, Brigham and Women's Hospital (Boston, MA, USA), under a protocol approved by the hospital's institutional review board for human research, and three postmortem brains (19, 21, and 22 GW) were provided by the Allen Institute Brain Bank (Seattle, WA, USA). Postmortem specimens were obtained with full parental consent, whereby the primary cause of death was complications of prematurity. The brains were grossly normal, and standard autopsy examination of all brains revealed minimal or no pathologic abnormalities at the macroscopic level. All research protocols were approved by the Institutional Review Board (IRB) at each institute.

All living participants (one 30 GW, one 34 GW, and three 40 GW) had clinically-indicated brain MRI studies that were diagnostically interpreted to show no abnormalities. Indications for imaging included concern for hypoxic ischemic injury, apnea, and transient choreiform movements after an upper respiratory tract infection. None had clinical concerns for a congenital malformation or genetic disorder. This *in vivo* part of this paper is a retrospective study of clinical MRI data, and the Institutional Review Board at Boston Children's Hospital deemed this an exempt project because the research is retrospective and involved existing data with no risk to patient confidentiality.

### Tissue preparation for HARDI

At the time of autopsy, all brains were immersion fixed and the brains from the Brigham and Women's Hospital were stored in 4% paraformaldehyde, and the brains from the Allen Institute Brain Bank were stored in 4% periodate-lysine-paraformaldehyde (PLP). During MR image acquisition, the brains from the Brigham and Women's Hospital were placed in Fomblin solution (Ausimont, Thorofare, NJ) (e.g., Takahashi et al., 2012) and the brains from the Allen Institute Brain Bank were placed in 4% PLP. These different kinds of solutions in which the brains from different institutes were placed tend to change the background contrast (i.e., we see dark background outside of the brain using Fomblin, and bright background using PLP), but they do not specifically change diffusion properties (e.g., FA and ADC) within the brain.

### Diffusion MRI procedures

The postmortem brain specimens from the Brigham and Women's Hospital were imaged using a 4.7T Bruker Biospec MR system (specimens from 17 GW to 31 GW) and specimens form the Allen Institute Brain Bank were imaged using a 3T Siemens MR system [three fetal (19, 20, 22 GW) specimens] at A. A. Martinos Center, Massachusetts General Hospital, Boston, MA. We used the 3T system as the brains from the Brain Bank were *in cranio* and did not fit in the 4.7T bore. To improve the imaging quality and obtain the best signal to noise and high spatial resolution, we used a custom-made MR coil with the 3T system that just fit the specimens. Different scanner systems were used to accommodate the different brain sizes, and magnetic resonance (MR) coils that best fit each brain sample were used to ensure optimal imaging.

For the brains from the Brigham and Women's hospital, a 3-dimensional (3-D) diffusion-weighted spin-echo echo-planar imaging (SE-EPI) sequence was used with a repetition time/echo time (TR/TE) of 1,000/40 ms, with an imaging matrix of 112 × 112 × 112 pixels. Sixty diffusion-weighted measurements (with the strength of the diffusion weighting, b = 8,000 s/mm^2^) and one non-diffusion-weighted measurement (no diffusion weighting or b = 0 s/mm^2^) were acquired with the duration of the diffusion gradients, δ = 12.0 ms, and the time interval between the start of the two diffusion gradients, Δ = 24.2 ms. The total acquisition time was ~2 h for each imaging session. The spatial resolution was 415 × 500 × 550 μm for the specimens at 20 and 21 gestational weeks; 440 × 520 × 720 μm for the specimen at 3 postnatal months. We determined the highest spatial resolution for each brain specimen with an acceptable signal-to-noise ratio of more than 130, and within a reasonable scan time of 2 h. For the brains from the Brain Bank, diffusion-weighted data were acquired over two averages using a steady state free precession sequence (TR/TE = 24.82/18.76 ms, α = 60°; 400 μm isotropic resolution). Diffusion weighting was isotropically distributed along 44 directions (b = 730 s/mm^2^) with 4 b = 0 images.

The brains of living patients were imaged on a 3T Siemens MR system, Boston Children's Hospital, Boston, MA. The diffusion pulse sequence used for imaging live participants was a diffusion-weighted spin-echo echo-planar imaging (SE-EPI) sequence, TR/TE 8320/88 ms, with an imaging matrix of 128 × 128 × 64 pixels. The spatial resolution was 2 × 2 × 2 mm. Thirty diffusion-weighted measurements (b = 1,000 s/mm^2^) and 5 non-diffusion-weighted measurements (b = 0 s/mm^2^) were acquired with δ = 40 ms and Δ = 68 ms.

### Diffusion data reconstruction for tractography

DiffusionToolkit and TrackVis (trackvis.org) were used to reconstruct and visualize tractography pathways. Tractography pathways were reconstructed using a HARDI model with a streamline/FACT algorithm and a 45° angle threshold. No threshold of fractional anisotropy (FA) was used for the fiber reconstruction. Brain mask volumes were used to terminate tractography structures instead of the standard FA threshold (Takahash et al., 2010; Takahashi et al., 2011, 2012, 2014; Song et al., 2015; Das and Takahashi, 2017; Vasung et al., 2017), because progressive myelination and crossing fibers in the developing brain can result in low FA values that may potentially incorrectly terminate tractography tracing in brain regions with low FA values.

### Tract delineation

Cortical, TC/CT, and CC pathways were identified in each subject by placing regions of interest (ROIs) in the cerebral cortex, thalami, and corpus callosum regions, referring to DTI and tractography atlases (Mori, 2007; Catani and Thiebaut de Schotten, 2008; Oishi et al., 2010). The size of all the ROIs were carefully optimized not to include other white matter pathways as well as not to miss the cortical, TC/CT, and CC pathways by changing the size and location several times. We also occasionally used additional ROIs to exclude clearly different pathways from the pathways of interest.

**Figure 1:**
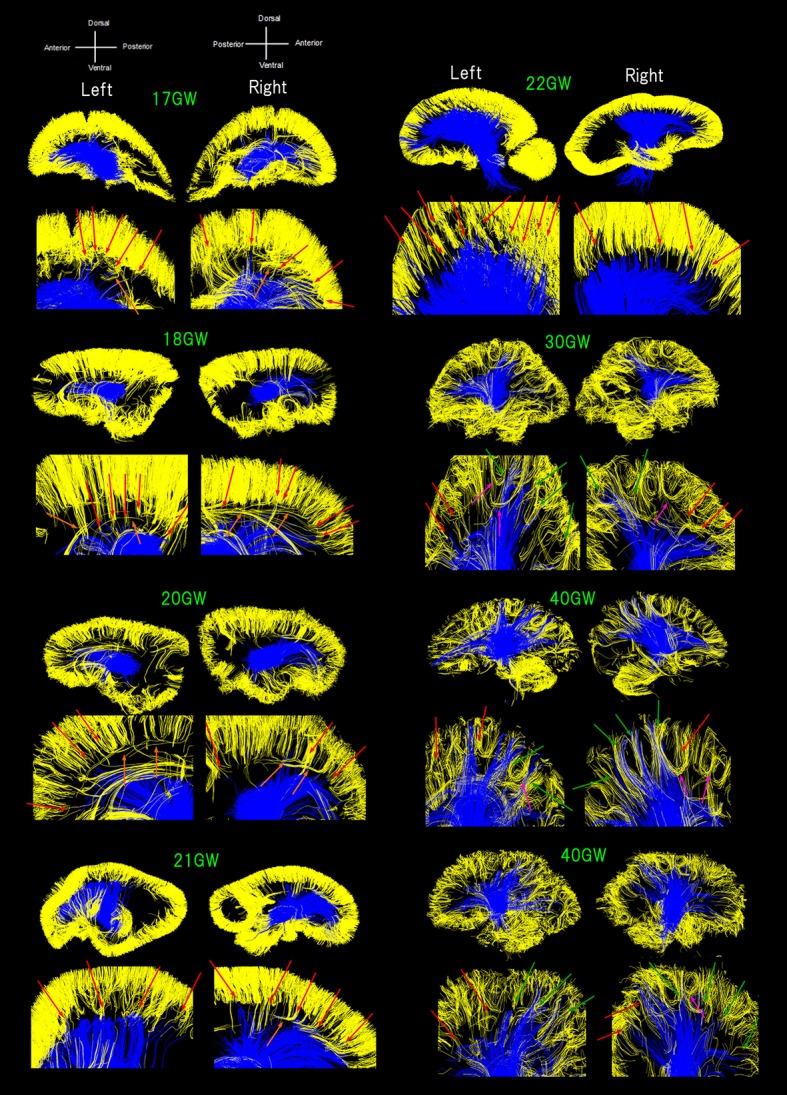
Sagittal overviews of all diffusion tractography pathways starting/ending in the thalami (blue pathways) and pathways within a sagittal plane starting/ending in the cortical plate (CP) in early developmental stages or within the gray matter cortex in later stages (yellow pathways). Ages range from 17 to 40 GW. Red arrows**:** pathways from the thalami converged with some pathways from the cerebral cortex as early as 17 GW, mainly in anterior and middle regions of the brain until 22 GW. Green arrows**:** posterior pathways from the thalami also converging with cortical pathways at 30 GW and onwards. Orange arrows**:** a few exceptional pathways turning perpendicularly, along with majority of short-range, straight cortical pathways. Pink arrows**:** several groups of fiber bundles at 30 GW and onwards, making u-shaped trajectories.

**Figure 2:**
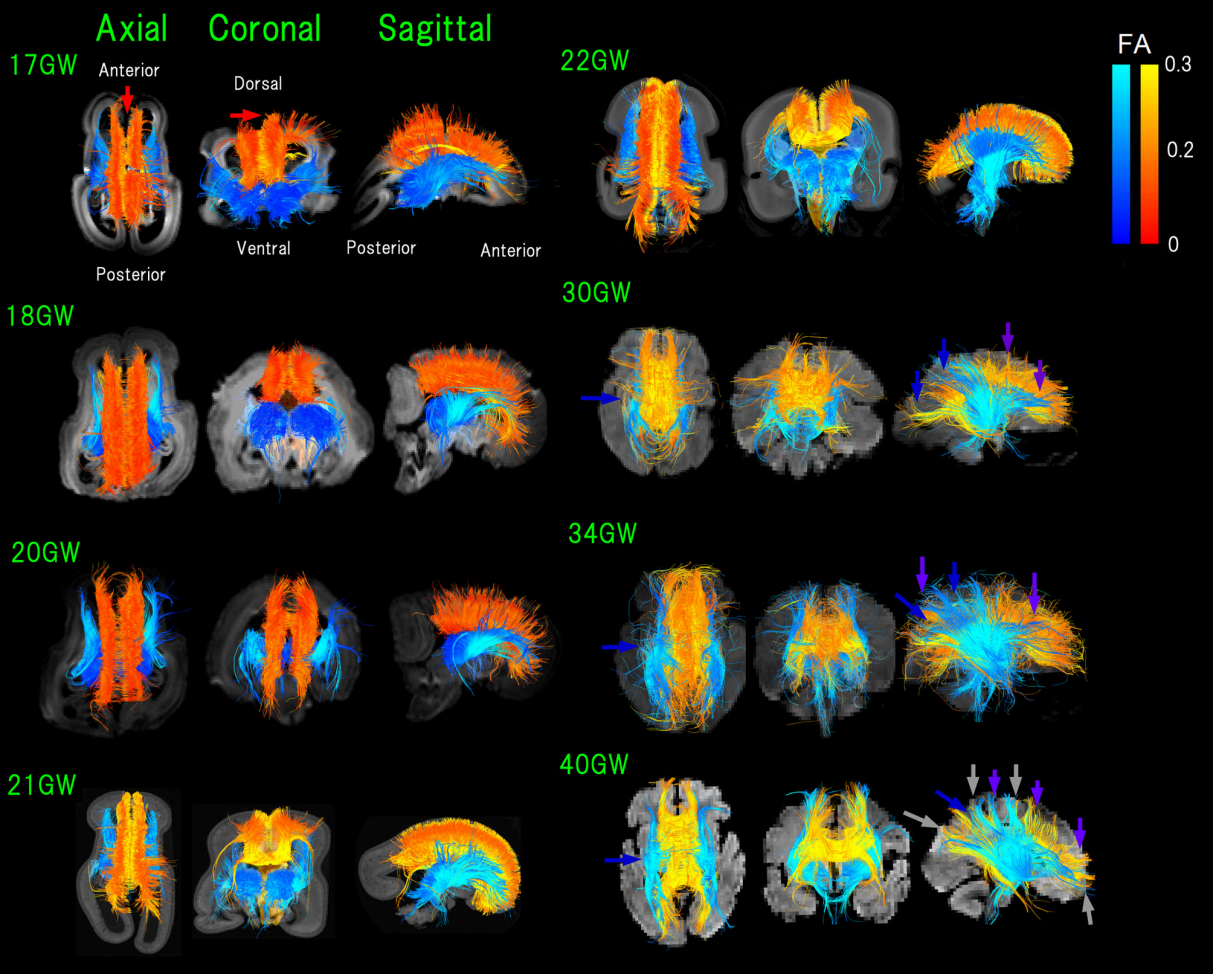
Figure 2. Overview of thalamocortical (TC, blue) and corpus callosal (CC, red-orange-yellow) pathways from 17 to 40 GW detected by diffusion tractography, color-coded by fractional anisotropy (FA) values. While it appears that some pathways lay outside of the brain surface, these are due to the angles of the images. Light-blue arrows**:** pathways linked to thalami that were greater in number compared to those in posterior brain regions (17–21 GW). Blue arrows**:** TC pathways in the middle and posterior brain regions increased and equivalent in number with pathways linked to thalami (22 GW-). Green arrows**:** increased anterior TC pathways reaching the cortex by 20 GW. Red arrows: CC pathways connecting bilateral medial walls of the brain observed fully penetrated into the upper edge of the cortical plate at 17 GW. By 30 GW, global TC and CC pathways reached the cortex (pink and purple arrows, respectively). Orange arrows: condensed groups of TC pathways identified by 30 GW. Gray arrows**:** TC and CC bands further being condensed and many CC and TC bands overlapping with each other as the two pathways converged at 40 GW.

The color-coding of tractography pathways in Figure 1 was determined for a visualization purpose: thalamocortical pathways in blue and cortical pathways in yellow. The color-coding of tractography pathways in Figure 2 was based on FA values (thalamocortical in red to yellow, and corpus callosal pathways in dark to light blue).

## Results

### Convergence of emerging thalamic and cortical pathways

Figure 1 shows all pathways starting/ending in the thalami (blue pathways) and pathways within a sagittal plane starting/ending in the CP in early developmental stages or within the gray matter cortex in later stages (yellow pathways; see Method section). Throughout development, TC pathways grew toward the cortex and cortical pathways descended toward the center of the brain. Some of the pathways from the thalami were found converged with some pathways from the cerebral cortex as early as 17 GW (Figure 1, red arrows). Such convergence was observed mainly in anterior and middle regions of the brain until 22 GW. At 30 GW and onwards, posterior pathways from the thalami also converged with cortical pathways (Figure 1, green arrows). A majority of the pathways linked to the thalami were terminated around the lower edge of the cortical pathways located within the cortical plate (CP) until 22 GW, with only a few pathways penetrating into the CP, while such long penetrating pathways were found more in number at and after 30 GW, and by term, these pathways were found at the upper edge of the cerebral cortex. The majority of short-range cortical pathways were straight until 22 GW with a few exceptional pathways turning perpendicularly (Figure 1, orange arrows). At 30 GW and onwards, several groups of fiber bundles were observed making u-shaped trajectories (Figure 1, pink arrows).

### Convergence of thalamocortical and corpus callosal pathways

In early fetal stages, pathways linked to thalami were greater in number compared to those in posterior brain regions (17–21 GW in Figure 2, light-blue arrows). Starting from 22 GW, TC pathways in the middle and posterior brain regions increased in number, equivalent with pathways linked to thalami (Figure 2, blue arrows). No TC pathways reached the upper edge of the CP in the middle of the brain at 22 GW. By 20 GW, more anterior TC pathways reached the cortex (Figure 2, green arrows).

At 17 GW, CC pathways connecting bilateral medial walls of the brain were observed already fully penetrated into the upper edge of the CP (Figure 2, red arrows). By 30 GW, these pathways had a higher FA and were thick and dense. Interestingly, when CC pathways still had relatively lower FA values (around 0.1 in red/orange at 18 GW), the center of the TC pathways already showed higher FA values (more than 0.2 in light blue).

By 30 GW, global TC and CC pathways reached the cortex (pink and purple arrows, respectively, in Figure 2). By 30 GW, condensed groups of TC pathways were identified and they continued to thicken until term (Figure 2, orange arrows). The same trend was observed in CC pathways after 30 GW, and at the same time, we observed that CC pathways became sparser than earlier stages. At 40 GW, TC, and CC bands further condensed and many CC and TC bands overlapped with each other as the two pathways converged (Figure 2, gray arrows).

## Discussion

Emerging cortical, thalamocortical (TC) and corpus callosal (CC) pathways of human postmortem fetal brains and *in vivo* subjects' brains ranging from 17 to 40 GW were successfully detected and studied using HARDI tractography. Descending cortical and ascending TC pathways were observed touching and running along each other as early as 17 GW. Many CC pathways reached the full length up to the cortical surface as early as 17 GW, while pathways linked to thalami (not only TC axons but also including pathways linked to thalamic neuronal migration) reached the cortical surface at and after 20 GW. These results suggest that CC pathways developed earlier than the TC pathways. Both TC and CC pathways were widespread at early stages, but projected to specific regions of the cortex and overlapped in some regions by 40 GW. Given that TC and CC pathways closely interact each other not only during development but also after maturation, it is important to better understand structural basis of their relationships during early brain development.

We previously detected pathways likely linked to neuronal migration from the ventricular zone and GE to the thalami (Wilkinson et al., 2016). Between the ventricular zone and thalami, more tractography pathways were found in anterior compared to posterior regions, which was in agreement with postnatal observations that the anterior TC segment had a higher track count and volume than the posterior segment. The neuronal migration pathways from the ventricle in the anterior thalamic regions were replaced with white matter-like pathways that did not terminate in the ventricular zone, running through deep white matter regions between 22 and 30 GW. This time frame is also when our study saw the convergence of the TC and CC pathways, suggesting that this period is crucial to the co-development of these pathways. Regarding the GE pathways, three differential pathways likely linked to neuronal migration were detected from the anterior GE to the middle part of the thalami, from the dorsal GE to the dorsal thalami, and from the mid-posterior GE to the mid-posterior thalami (Wilkinson et al., 2016). It is possible that some of these pathways were included as the detected pathways that “handshake”; pathways between the thalamus and the GE in this study. Unfortunately, it is challenging to determine using HARDI whether the detected pathways are corresponding to neuronal migration pathways or if they have already been replaced by axonal pathways.

This study showed that the onset of the TC pathway development was more delayed than that of the CC pathway, and the anterior TC pathway that extended to the frontal cortex developed first. It is also shown that the middle and anterior parts of the TC pathway that connect to other areas like the somatosensory cortex and the visual area developed later. While CC pathways became more sparse and appeared to be pruning after 22 GW, the TC pathway seemed to be thickening and began extending at around 20 GW which continued to term. Both the TC and the CC pathways were found to form condensed groups of pathways that target specific areas of the cortex, and many of these groups followed similar trajectories. While one of our past research studies (Cohen et al., 2016) showed that the CC and TC pathways took similar postnatal growth patterns, the current results suggest that in earlier developmental stages, the CC structurally matures first.

Although the TC and CC projection pathways are closely connected throughout development and are involved in many of the same systems, their reactions to critical damage are seemingly different (Miller et al., 1991). Following the formation of lesions of the thalamus in hamsters, rerouting of pathways and altering of previously existing typical pathways were found. One alteration was the addition of CC pathways coming from the temporal cortex, which are not seen in the control group. Compared to TC pathways, CC pathways have the unique ability to reorganize after damage. Miller and colleagues have hypothesized that TC pathways may be unable to alter after damage because they have developed further at this point than CC pathways have, allowing CC projections to have more plasticity further into development. Other studies have shown that disturbances with either or both of these pathways can coincide with multiple disorders, including schizophrenia (Mitelman et al., 2009; Marenco et al., 2012; Collinson et al., 2014; Klingner et al., 2014), autism (Gozzi et al., 2012; Nair et al., 2013; Hanaie et al., 2014), and bipolar disorder (Anticevic et al., 2014; Yamada et al., 2015). Callosal dysfunction can also lead to epilepsy (Andrade et al., 2014), major depressive disorder (Cyprien et al., 2014), obsessive-compulsive disorder (Jose et al., 2015) and subcortical ischemic vascular dementia (Wu et al., 2015). Not only does damage to the pathways themselves result in a number of disorders and impairments, but environmental exposures, including visual stress, can result in negative effects on the TC pathway, leading to abnormal interactions of the CC pathway (Yinon and Hammer, 1990). Yinon and Hammer provided evidence that the TC pathways are necessary for CC pathways to accurately transfer visual information between hemispheres. Another study supporting the necessary functional interactions between TC and CC pathways was performed by Pallas et al. (1999). They rerouted TC pathways in deaf-induced ferrets, which resulted in the ferrets having altered CC connections, supporting the adaptation of one pathway in response to damage of the other, in order to decrease the injurious effects.

Development of TC pathways to term suggests implications on TC pathways in the birth of premature infants. Ball et al. (2013) used tractography to establish the TC connectome of both infants born at term and premature infants, born between 23 and 34 GW. Their study provides evidence that preterm birth has a detrimental impact on TC pathways, with a decrease in TC connections to multiple brain regions, including the supplementary motor areas, occipital lobe, temporal gyri, and frontal cortices. As premature infants are born before the TC pathways have fully developed, it is no surprise that preterm birth is associated with an increased risk of multiple developmental disorders, including problems with language, attention, memory, behavior, and learning.

The current study is evidence that HARDI tractography is able to determine the development patterns of TC and CC pathways from gestational development, continuing into the postnatal time frame. Not only can researching the development patterns of the TC and CC pathways individually help further our understanding of brain development, but studying the TC and CC pathways together will help develop an understanding of the networks these pathways are involved in. Understanding human brain development at both specific and broad ranges, will lead to appropriate pre- and post-natal care for early detection and treatment of developmental disorders.

The cortical pathways in Figure 1 were identified using ROIs for the cortex and the GE. For Figure 2, CC pathways were identified using ROIs in the corpus callosum. Therefore, we believe we did not show contaminated radial pathways in this paper. Potential limitations of this study include the small number of specimens across gestational ages, the use of multiple MRI acquisition systems, and the nature of diffusion tractography. The limited number of specimens available in this analysis (one per developmental stage) would impose substantial uncertainty to any reported quantitative findings and so we have relegated this topic to future work. We believe that the issue of different acquisition systems is not significant as tractography of both systems yielded similar trends. Given the nature of diffusion tractography, even with HARDI, it is possible that the pathways observed to terminate within the GE structure may be due to strong coherent tissue structures in the GE (e.g., Miyazaki et al., 2016). However, this would not be the case, because even at 17 GW, when the GE is still strongly coherent, several pathways from the thalamus reached at the bottom of the cortical plate running through the GE.

Partial volume effects with limited spatial resolution of diffusion MRI could cause tractography pathways erroneously terminating at the brain surface. However, in our data, there are many tractography pathways that terminate before reaching the upper layer of the cortical mantle (e.g., Vasung et al., 2017). Another explanation for tractography pathways that seem to terminate at the brain surface is that the location of the anatomical image is not always in-plane with the tractography pathways shown in the figures. Since tractography pathways are 3-dimensional, selecting one structural plane to overlay the pathways on it could cause misunderstanding for the spatial relationships between tractography pathways and anatomical image.

Since our results may indicate that CC pathways develop earlier than TC pathways, and TC pathways seem to follow the CC pathways and converge with them in late gestational ages, it is possible that CC pathways are influential in the TC development. However, it is also possible that the TC pathways find their destinations independently from CC pathways. It would be necessary to study this topic in patients with callosal agenesis to answer this question, a study which is currently underway in our group.

Although it was not always clear from the data whether the brain regions where “condensed” TC and CC pathways were observed are corresponding to the locations of gyral structures, the borders of visual areas where TC and CC pathways are supposed to converge have not been linked to gyral curvatures in the literature (e.g., Caspers et al., 2015). It would be interesting to study in more detail the locations of convergent regions of TC and CC pathways in human brains during development in the future.

## Author contributions

MW and ET designed the study. ET obtained the data. RW, MW, TK, and ET processed the data. MW, TK, and ET analyzed and wrote the paper.

### Conflict of interest statement

The authors declare that the research was conducted in the absence of any commercial or financial relationships that could be construed as a potential conflict of interest.
